# Male Smokers' and Non-Smokers' Response Inhibition in Go/No-Go Tasks: Effect of Three Task Parameters

**DOI:** 10.1371/journal.pone.0160595

**Published:** 2016-08-08

**Authors:** Xin Zhao, Xiaoting Liu, Xiangyi Zan, Ge Jin, Joseph H. R. Maes

**Affiliations:** 1 Behavior Rehabilitation Training Research Institution, School of Psychology, Northwest Normal University, Lanzhou, 730070, China; 2 Lanzhou University Second Hospital, Lanzhou, 730070, China; 3 College of Education, Lanzhou City University, Lanzhou, 730070, China; 4 Donders Institute for Brain, Cognition and Behaviour, Centre for Cognition, Radboud University, PO Box 9104, Nijmegen, 6500 HE, The Netherlands; University of Pennsylvania, UNITED STATES

## Abstract

Impaired response inhibition plays a major role in many addictive behaviors. However, in studies using go/no-go tasks, findings regarding the presence of response inhibition deficits in nicotine-dependent individuals are mixed. This might be due to differences between studies on a number of task parameters. Here we aimed to identify task conditions under which go/no-go task performance deficits can be observed in smokers and to characterize the nature of such deficits. Sixty-one male students (30 smokers, 31 non-smokers) performed a go/no-go task while independently manipulating three task parameters: (1) percentage no-go trials (50% or 25%), (2) stimulus presentation time (600 ms or 200 ms), and (3) nature of no-go stimuli (cigarette related or cigarette unrelated). Three measures, reaction time on go trials and percentage correct responses on go and no-go trials, served as performance indicators. Under 200-ms but not 600-ms stimulus presentation conditions, the smokers responded faster on go trials and made more errors on both go and no-go trials than the non-smokers did. These differences occurred irrespective of the percentage of no-go trials and nature of no-go stimuli. The accuracy differences disappeared after controlling for the response time differences, suggesting a strong speed-accuracy trade-off. This study contributes to unraveling the conditions under which smokers display impaired inhibition performance and helps to characterize the nature of this impairment. Under task conditions prompting fast responding, smokers are more prone to increase response speed and to make more errors than non-smokers.

## Introduction

Previous studies suggest that at least some forms of substance abuse and addiction are associated with increased impulsivity in general, and impaired behavioral inhibition, one of the major components of executive functioning [1], in particular [2]. These deficits are subserved by a frontostriatal circuitry [3]. These behavioral and neural associations have also been suggested to hold for nicotine/tobacco use (smoking) (e.g., [4–7]). However, as outlined below, evidence for this suggestion is mixed, especially when considering studies that used go/no-go tasks for measuring inhibition.

In go/no-go tasks, participants are asked to respond (e.g., with a button press) to a specific stimulus or a class of stimuli (‘go’ trials) but to refrain from responding upon being presented with another stimulus or class of stimuli (‘no-go’ trials). Usually, the participant is instructed to respond as fast as possible to go stimuli, while maintaining accuracy as much as possible. In one version of this task, the sustained attention to response task (SART) [8], the percentage of go trials is lower than, or equal to that of no-go trials. However, especially in the context of measuring inhibitory capacities, the task contains many more go than no-go trials. In this way, a potent behavioral response tendency is induced that has to be inhibited occasionally when a no-go stimulus is presented. Various indices can be calculated from this task, most of which have been shown to be associated with disorders such as ADHD [9] and major depression [10], as well as with substance abuse and addiction [2].

The three most commonly used dependent measures derived from go/no-go tasks are the mean response time on go trials (hereafter: MRT), percentage of omission errors (OEs; not responding to go stimuli), and percentage of commission errors (CEs; incorrectly responding to no-go stimuli). The MRT measure is assumed to reflect a variety of cognitive processes, but primarily vigilance and decision-making speed. At least in go/no-go task versions with a relatively small number of go trials, OEs are assumed to reflect lapses of attention or vigilance [11] or task disengagement. CEs are generally assumed to reflect response disinhibition or impulsivity, and CE rate is the most commonly used inhibition index from the task. However, these measures may not be independent. Specifically, a clear speed-accuracy trade-off favoring speed would implicate short RTs, in turn leading to high rates of OEs and especially CEs (and vice versa for a trade-off favoring accuracy). Therefore, statistically controlling for speed-accuracy trade-offs might increase the validity of this type of task [12].

Studies comparing go/no-go task performance in smokers and non-smokers have revealed mixed results. For example, two studies [13, 14] found that smokers displayed more CEs than non-smokers, but observed no group difference on the other two measures (MRT and OEs). However, three other studies failed to observe a significant difference on any of the three performance measures [15–17]. These mixed results might be due to differences between studies on a number of variables, such as the degree of nicotine dependence, as estimated with questionnaires, ranging from weak [15] to medium [13], and hours of abstinence from smoking in the smokers’ group before testing, varying from 0 h (ad lib smoking) [14] to at least 8.5 h [17]. These are important differences because they implicate differences in the balance between possible acute effects of nicotine and nicotine withdrawal effects on cognitive task performance (e.g., [18, 19]). However, differences between studies on three additional task-related parameters are of particular interest for the purpose of the present study. The first is the percentage of no-go trials. The percentage of no-go trials was equal to the percentage of go trials in one study [16] whereas the no-go trial percentage in the other studies ranged from 9% [17] to 25% [13]. The second parameter concerns the stimulus presentation time and/or the response-time window (or inter-stimulus interval; ISI). For example, one study used an 800-ms stimulus presentation time,[17], whereas in another study [13] the presentation time was 200 ms, followed by a 1020‒1220 ms empty interval during which the participant could respond. Two studies did not present clear information concerning this parameter [15, 16]. A third parameter concerns the nature of go- and no-go stimuli. These consisted of either geometrical figures [16], single letters [15], a sequence of letters [14, 17], or cigarette-related or -unrelated pictures [13]. The ‘value’ on these task parameters in isolation and/or in specific combinations might either be, or not be, favorable for observing performance differences between smokers and non-smokers. For example, especially ‘difficult’ task conditions, implicating a strong challenge of inhibition capacity, such as when using a low no-go probability, a short stimulus-presentation time and response window, and cigarette-related stimuli as no-go stimuli, might be particularly conducive for observing impaired task performance in smokers relative to non-smokers.

Given the mixed previous results, the purpose of the present study was to identify the boundary conditions for observing go/no-go task performance impairments in smokers relative to non-smokers. Moreover, we aimed to further characterize the nature of these impairments in terms of the three outcome measures outlined above. To this end, we performed a parametric investigation into the effect of three go/no-go task parameters: (1) percentage of no-go trials (50% or 25%), (2) stimulus presentation duration (600 ms or 200 ms, with a fixed 1000 ms ISI), and (3) nature of no-go stimuli (cigarette related or cigarette unrelated). These conditions were all presented under an identical nicotine abstinence condition for the smokers. Our overall working hypothesis was that a difference between smokers and non-smokers would be especially found for CEs under the conditions of a low percentage of no-go stimuli, a short stimulus presentation time, and cigarette-related stimuli as no-go stimuli.

## Materials and Methods

### Participants

Sixty-three male students (30 smokers, 33 non-smokers) were recruited at the university campus through posters. The participants’ age ranged from 18 to 25 years. Two non-smokers were excluded due to their misunderstanding of the task instructions. The final group consisted of 30 smokers and 31 non-smokers. Smokers fulfilled the following criteria (checked with a short questionnaire specifically designed for use in this study): smoking ≥ 10 cigarettes/day, smoking regularly for ≥ 2 years, no current use of medications that might affect cognition, no medical or psychiatric condition that could affect brain function, age ≥ 18 years, no history of head trauma, smoking ≤ 1 marijuana cigarette per week, using ≤ 10 standard drinks of alcohol per week, and no regular use of substances of abuse other than alcohol or marijuana. There were large individual differences among the smokers in level of nicotine dependence, as assessed by the Fagerström test for nicotine dependence (FTND; see below; range = 1–8; the number of participants with a low, moderate, and strong dependence was 9, 9, and 12, respectively). Non-smokers had no current or previous history of cigarette smoking and fulfilled all other non-smoking related criteria outlined for the smokers. They all had an FTND score of 0. All participants had normal or corrected-to-normal vision. The present study was approved by the Northwest Normal University Ethics Committee and participants signed consent forms after being informed about the general nature of the questionnaires and the task to be performed. The study was conducted according to the principles expressed in the Declaration of Helsinki. Participants were paid a small amount of money as remuneration. They also completed questionnaires assessing demographic information, medical history, smoking history, nicotine dependence, and presence of symptoms of anxiety. The latter questionnaire was included because previous studies suggest that anxiety is a personality feature that might affect go/no-go task performance [20]. The two groups were fully matched on the various measures except for the smoking-related variables (see Table 1).

**Table 1:** Demographic information and questionnaire scores for smokers and non-smokers (mean and SD).

	Smokers (n = 30)	Non-smokers (n = 31)
Age	21.40 (2.14)	21.13 (1.20)
Height	172.33 (4.78)	172.77 (4.39)
Weight	124.93 (20.01)	124.84 (11.57)
Cigarettes/day	16.80 (2.51)	0
Fagerström score	4.57 (2.13)	0
Beck Anxiety Inventory score	63.54 (26.52)	63.20 (21.12)

### Material

#### The Fagerström Test for Nicotine Dependence (FTND)

The Fagerström test for nicotine dependence (FTND) [21] is a six-item questionnaire to measure nicotine dependence. An overall score on the FTND ranges from 0 to 10. A score of 1–3 indicates low dependence, 4–5 indicates moderate dependence, and ≥ 6 indicates strong dependence. We used the revised Chinese version of the FTND in our study, which has proved to be valid and reliable [22].

#### The Beck Anxiety Inventory (BAI)

The Beck Anxiety Inventory [23] is a self-rating scale to assess anxiety level. The scale consists of 21 items that can be responded to using a 4-point Likert scale. The Chinese version of the BAI that we used has a good reliability and validity [24].

#### Go/no-go task

The material used in the go/no-go task consisted of 54 color pictures selected from the International Affective Picture System (IAPS) [25] and internet; half of the pictures were cigarette related. The pictures were edited using Photoshop 7.0, to ensure that they were all in 210 × 210 pixel form. We added a blue and yellow frame to all cigarette-related and cigarette-unrelated pictures, respectively. After the go/no-go task, the subjects evaluated each of the 54 pictures on pleasantness, arousal, dominance, and relatedness to cigarettes, using a 9-point Likert scale. The pictures were presented in two 27-picture blocks using Eprime 2.0. Pictures were presented for 5000 ms in a random sequence on a Lenovo 17- inch screen, with a screen resolution of 1444 × 900, and a vertical refresh rate of 75.1 Hz. A Group × Picture Type (cigarette related or unrelated) analysis of variance (ANOVA) on the scale measuring cigarette relatedness only revealed that cigarette-related pictures (*M* = 7.45, *SD* = 1.66) were rated significantly higher than non-cigarette-unrelated pictures (*M* = 2.76, *SD* = 1.48), *F*(1, 59) = 1 84.56, *p* < 0.001, *η*^*2*^ = 0.76. An identical ANOVA on the pleasantness, arousal, and dominance ratings did not reveal any significant effects, *F*s < 3.19, *p*s > .08, *η*^2^s < .05.

**Fig 1:**
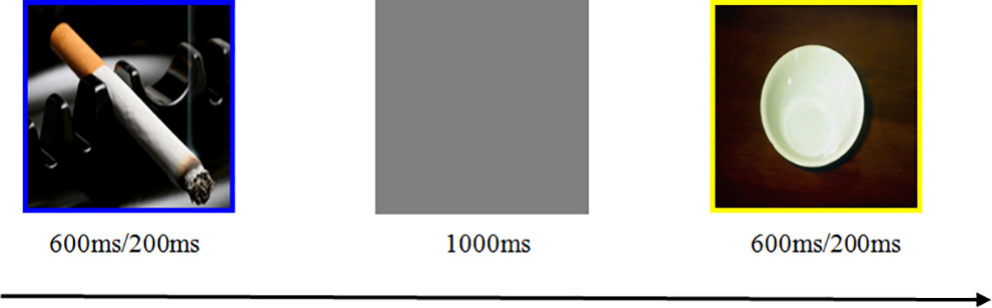
Example of a trial with a cigarette-related and cigarette-unrelated picture in the go/no-go task.

The task, also programmed in Eprime 2.0 and shown on the 17-inch screen as used for the ratings, comprised the following four difficulty levels, manipulating stimulus presentation time (1^st^ value) and percentage no-go stimuli (2^nd^ value): Level 1: 600 ms/50%, Level 2: 600 ms/25%, Level 3: 200 ms/50%, and Level 4: 200 ms/25%. Each picture had a blue or yellow frame (see Fig 1 for an example of a trial with a cigarette-related and cigarette-unrelated stimulus, respectively). The frame color indicated whether a stimulus corresponded to a go or a no-go trial. Each stimulus was followed by a grey screen for 1000 ms. Participants were instructed to respond to each go stimulus by pressing the letter J on the keyboard with the right index finger as fast as possible, and to withhold their response to no-go stimuli. They were explicitly instructed to maintain accuracy throughout the task. Before the start of each set of difficulty-level blocks (see hereafter), participants received 20 practice trials involving two cigarette-related and two cigarette-unrelated pictures that were not used in the remainder of the task. During the first 10 practice trials, no-go trials were indicated by cigarette-unrelated pictures; cigarette-related pictures served as no-go stimuli during the second 10-trial practice block. The proportion of no-go trials during these practice trials was in accordance with the corresponding proportion of no-go trials during the subsequent difficulty-level blocks. Participants proceeded with the corresponding difficulty level of the main experiment once their practice accuracy rate exceeded 85%. Each difficulty level within the main task comprised four 100-trial blocks. During each of the first two blocks, go trials were indicated by pictures with a blue frame; on each of the last two 100-trial blocks, go-trials were indicated by the yellow-framed pictures. This design implicates that, for each difficulty level, half of the no-go stimuli were cigarette related and blue-framed; the other half were cigarette unrelated and yellow-framed. Within each block, each of the 25 cigarette-related and 25 cigarette-unrelated pictures used was presented 1‒3 times, depending on the percentage of no-go trials. Participants had a short break between blocks. The four difficulty levels were presented in a random order for each participant. Each level took approximately 10 min to complete, depending on the length of the breaks.

### Procedure

To reduce acute nicotine effects as well as withdrawal effects, the smokers had been asked to abstain from smoking for at least one hour before initiation of the experiment. Upon arrival at the laboratory, participants signed the informed consent forms and completed the questionnaires. The participant was then seated at a distance of 60 cm in front of the centre of the laptop screen and completed the go/no-go task. Finally, the participant rated the pictures used on the different scales. The entire experiment took approximately 60 min.

### Data analysis

Trials with a MRT exceeding two standard deviations were excluded (< 15% of each participant’s trials). We used the MRT and accuracy (proportion of trials with a correct response) on go and no-go trials as outcome measures. Each of these measures was subjected to a repeated measures analysis of variance (RM-ANOVA), with Group (smokers vs. non-smokers) as between-subject factor, and no-go percentage (hereafter: Percentage; 50% versus 25%) and Picture Type (cigarette-related versus cigarette-unrelated pictures) as within-subject factors. Significant interactions were followed up by simple main effect analyses. The RM analyses were performed separately for the two stimulus presentation duration (600- ms and 200-ms) blocks. This separation was based on the outcome of preliminary RM-ANOVAs that incorporated presentation time as additional within-group factor. These analyses revealed a significant Group × Presentation Time × Percentage interaction for MRT and go accuracy thereby motivating a separate Group × Picture Type × Percentage ANOVA for each stimulus presentation time condition. Because the data for the 200-ms presentation time conditions suggested a strong speed-accuracy trade-off (see below) we also performed analyses of covariance (ANCOVAs) in which we controlled for MRT when evaluating group differences on the accuracy measures [12]. A *p* value of < .05 was adopted as criterion for statistical significance in all analyses.

## Results

### Trials with 600-ms stimulus presentation

#### MRT

**Fig 2:**
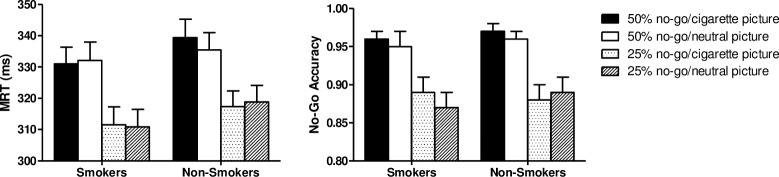
Left: Mean reaction time (+ SEM) of smokers and non-smokers on go trials of the 600-ms stimulus presentation time blocks. Values are presented separately for each no-go stimulus percentage and stimulus type condition. The only significant effect was faster overall responding on the 25% no-go trials condition than the 50% condition (*p* < 0.001). Right: Mean **(+** SEM) proportion of trials of the 600-ms stimulus presentation time blocks on which smokers and non-smokers correctly omitted responding to the no-go stimulus, separately for each no-go stimulus percentage and stimulus type condition. The only significant effect was a higher accuracy rate for the 50% no-go trials condition than the 25% condition (*p* < 0.001).

The MRT for each group, picture type, and no-go trial percentage is displayed in the left panel of Fig 2. ANOVA revealed a main effect of Percentage, *F*(1, 59) = 41.80, *p* < 0.001, *η*^*2*^ = 0.42, reflecting that participants were slower to respond on trial blocks with 50% no-go trials (*M* = 334.47, *SD* = 30.40) than on blocks with 25% no-go trials (*M* = 314.63, *SD* = 29.69). All other main and interaction effects were not significant, *F*s < 1.

#### No-go trial accuracy

The right panel of Fig 2 shows the mean proportion of correct non-responses on no-go trials, separately for each group, picture type, and no-go trial percentage condition. ANOVA on these data again only revealed a main effect of Percentage, *F*(1, 59) = 72.48, *p* < 0.001, *η*^*2*^ = 0.55; other *F*s < 1.82. Participants were more accurate on trial blocks with 50% no-go trials (*M* = 0.96, *SD* = 0.06) than on blocks with 25% no-go trials (*M* = 0.88, *SD* = 0.10).

#### Go trial accuracy

The accuracy on go trials was 100% for each participant in each of the conditions.

### Trials with 200-ms stimulus presentation

#### MRT

**Fig 3:**
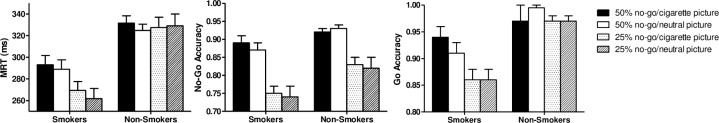
Left: Mean reaction time (+ SEM) of smokers and non-smokers on go trials of the 200-ms stimulus presentation time blocks. Values are presented separately for each no-go stimulus percentage and stimulus type condition. Overall, smokers responded faster than non-smokers (*p* < 0.001), and the 25% no-go trial condition elicited faster responding than the 50% condition (*p* = 0.01), especially for smokers (*p* < 0.001). Middle: Mean **(+** SD) proportion of trials of the 200-ms stimulus presentation time blocks on which smokers and non-smokers correctly omitted responding to the no-go stimulus. Overall, non-smokers were more accurate than smokers (*p* = 0.001) and response accuracy was larger in the 50% no-go trial condition than the 25% condition (*p* < 0.001). Right: Mean **(+** SD) proportion of trials of the 200-ms stimulus presentation time blocks on which smokers and non-smokers responded to the go stimulus. Overall, non-smokers were more accurate than smokers (*p* = 0.001) and response accuracy was larger in the 50% no-go trial condition than the 25% condition (*p* < 0.001), especially for the smokers (*p* < 0.001).

The MRTs on the 200-ms presentation time trials for each group and condition are presented in the left panel of Fig 3. ANOVA revealed a main group effect, *F*(1, 59) = 21.87, *p* < 0.001, *η*^*2*^ = 0.27, reflecting that, overall, the smokers responded faster (*M* = 278.31, *SD* = 47.34) than the non-smokers (*M* = 328.30, *SD* = 34.57). The main effect of no-go trial percentage was also significant, *F*(1, 59) = 8.34, *p* = 0.01, *η*^*2*^ = 0.12, with the RT in the 50% condition (*M* = 309.65, *SD* = 45.28) being longer than in the 25% condition (*M* = 296.95, *SD* = 61.41). There was also a significant Group × Percentage interaction, *F*(1, 59) = 8.40, *p* = 0.01, *η*^*2*^ = 0.13. Subsequent simple main effect analyses revealed a significant difference in MRT between the 50% condition (*M* = 291.04, *SD* = 46.94) and the 25% condition (*M* = 265.58, *SD* = 47.92) for the smokers, *F*(1,29) = 16.25, *p* < 0.001, *η*^*2*^ = 0.36, but not for the non-smokers (50% condition: *M* = 328.27, *SD* = 34.79; 25% condition: *M* = 328.32, *SD* = 57.15), *F* < 1. For each of the no-go trial percentage conditions, the MRT was significantly lower for smokers than non-smokers, *F*(1,59) = 13.77, *p* < 0.001, *η*^*2*^ = 0.19, for the 50% condition, and *F*(1,59) = 23.64, *p* < 0.001, *η*^*2*^ = 0.29, for the 25% condition. All other main and interaction effects from the overall Group × Picture Type × Percentage ANOVA were not significant, *F*s(1, 59) < 2.06, *p*s > .16, *η*^*2*^s < 0.03.

#### No-go trial accuracy

ANOVA on the no-go trial accuracy data (see middle panel Fig 3) revealed a main effect of group, *F*(1, 59) = 7.06, *p* = 0.01, *η* ^*2*^ = 0.11. The smokers’ accuracy (*M* = 0.81, *SD* = 0.13) was lower than that of the non-smokers (M = 0.88, *SD* = 0.12). However, when controlling for MRT (pooled over the no-go percentage and picture type conditions) using ANCOVA, this group effect disappeared, *F* < 1. The main effect of no-go trial percentage was also significant, *F*(1, 59) = 111.95, *p* < 0.001, *η*^*2*^ = 0.66. The response accuracy on trials with 50% no-go trials (*M* = 0.90, *SD* = 0.08) was higher than that on trials with 25% no-go trials (*M* = 0.78, *SD* = 0.14). This main effect of percentage remained significant even after controlling for MRT, *F*(1, 58) = 7.83, *p* = 0.007, *η*^*2*^ = 0.12. All other main and interaction effects were not significant, *F*s(1, 59) < 1.60, *p*s > 0.21, *η*^*2*^s < 0.04.

#### Go trial accuracy

The right panel of Fig 3 displays the accuracy scores for each group, picture type, and percentage condition. ANOVA on these data revealed a main effect of group *F*(1, 59) = 16.91, *p* < 0.001, *η*^*2*^ = 0.22, reflecting a lower accuracy for smokers (*M* = 0.86, *SD* = 0.14) than non-smokers (*M* = 0.97, *SD* = 0.10). However, this group effect was no longer significant after controlling for overall MRT, *F*(1, 58) = 2.16, *p* = 0.15, *η*^*2*^ = 0.04. The main effect of no-go trial percentage was also significant, *F*(1, 59) = 9.48, *p* = 0.003, *η*^*2*^ = 0.14, with accuracy in the 50% condition (*M* = 0.95, *SD* = 0.13) being higher than in the 25% condition *(M* = 0.91, *SD* = 0.11). However, this main effect too became insignificant after controlling for MRT, *F* < 1. Except for the Group × Percentage interaction, *F*(1, 59) = 4.41, *p* = 0.04, *η*^*2*^ = 0.07, all other main and interaction effects were not significant, *F*s(1, 59) < 1.96, *p*s > 0.17, *η*^*2*^s < 0.04. The significant interaction reflected a significantly higher accuracy in the 50% condition (*M* = 0.93, *SD* = 0.12) than in the 25% condition (*M* = 0.86, *SD* = 0.12) for the smokers, *F*(1, 29) = 15.80, *p* = 0.001, *η*^*2*^ = 0.35, whereas there was no significant difference between the 50% (*M* = 0.98, *SD* = 0.13) and 25% (*M* = 0.97, *SD* = 0.06) conditions for the non-smokers, *F* < 1. The smokers performed less accurately than the non-smokers in both the 50% condition, *F*(1, 59) = 4.46, *p* = 0.04, *η*^*2*^ = 0.07, and the 25% condition, *F*(1, 59) = 25.25, *p* < 0.001, *η*^*2*^ = 0.30. However, the significant Group × Percentage interaction that motivated these simple main effect analyses became insignificant after controlling for overall MRT, *F*(1, 58) = 2.75, *p* = 0.10, *η*^*2*^ = 0.05.

## Discussion

In this study, we compared go/no-go task performance in smokers and non-smokers while manipulating three task parameters. We found that under 600-ms stimulus presentation time conditions, smokers did not differ from non-smokers on any of the three examined outcome measures. However, under a 200-ms presentation time condition, smokers made significantly more errors on both go and no-go trials. Moreover, at least with respect to errors on go trials, the smoker were more sensitive to the effect of the percentage of no-go trials. That is, their accuracy on go trials even dropped further with a decrease in no-go trial percentage from 50% to 25% whereas no such drop was observed for non-smokers. However, these between-group accuracy differences seemed to be entirely driven by between-group differences in RT to go trials. Under the 200-ms presentation condition, smokers were faster to respond on go trials than non-smokers, with the largest RT difference being observed under the 25% no-go trial condition. When controlling for overall MRT on go trials, the accuracy differences between groups became insignificant. These results suggest a strong speed-accuracy trade-off in both smokers and non-smokers. Increased speed came at the cost of decreased overall accuracy and especially smokers increased their response speed with increasing task demands (short presentation times and low no-go probability), which was associated with the occurrence of relatively many errors. A final notable finding was the absence of any differential effects as a function of the nature of the no-go stimuli, that is, whether they were cigarette related or unrelated.

The lower accuracy rates for go and no-go trials for the smokers are in accordance with the results reported in Luijten et al. [13]. In this study, a 200-ms stimulus presentation time was employed too, in combination with about the same ISI as used in our study, and a 25% no-go stimulus probability. Smokers were found to make more errors on both go and no-go trials (but especially no-go trials) than non-smokers. However, no between-group difference was found for MRTs. Perhaps, group differences on the accuracy rates would also have been diminished in this study if RT outliers had been removed and RTs had been used as a covariate. A lower no-go accuracy for smokers was also found in the study by Nestor et al. [14], but no MRT difference. However, there are important differences between this and our study in terms of, for example, abstinence before testing (ad lib smoking vs. 1 h abstinence), nature of stimuli, percentage of no-go trials, and stimulus presentation times. These differences complicate a direct comparison between the studies. This also holds for a comparison with the studies mentioned in the introduction that all reported null results for the difference between smokers and non-smokers on each of the outcome measures. Each of these studies differs on at least one of the variables of pre-test abstinence, nature of stimuli, stimulus presentation time, no-go stimulus probability, or nicotine dependency level.

The absence of main and interaction effects involving the stimulus type factor (cigarette related vs. unrelated) is unexpected in view of the abundant literature on attentional biases to addiction-related cues that have been shown to occur for many different types of addictive behaviour. Specifically, substance users’ attention tends to be strongly drawn towards stimuli that are relevant for their addiction. This attentional bias is assumed to be reciprocally related to both craving for the drug and impulsivity or impaired inhibitory control (see [26], for a review). In the present task, this would implicate that the smokers should be focussed more on cigarette-related than cigarette-unrelated cues. Moreover, in case of no-go cigarette-related cues they should have greater difficulty inhibiting a response to it (potentially mediated by induced craving) than would be the case for cigarette-unrelated no-go stimuli. Hence, the fact that we did not find a larger inhibition deficit for cigarette-related than cigarette-unrelated no-go stimuli runs counter to these expectations. However, the absence of this effect is consistent with the corresponding null results reported in a study performed by Luijten et al. [13]. As also noted by Luijten et al., these null results might be due to the cigarette-related stimuli, which were already presented during the practise phase and consistently presented in the first trial blocks in our study, and randomly intermingled with cigarette-unrelated pictures in Luijten et al.’s study, eliciting a craving for nicotine that, in turn, caused a general decrease in inhibitory control capacity. This general effect, then, affected responding to both cigarette-related and -unrelated no-go stimuli. However, this speculation clearly demands further experimental validation.

### Limitations and future directions

The design of the present task did not include a separate manipulation of stimulus presentation time and time available for responding (‘response window’). The 600- and 200-ms presentation time conditions also implied a difference in response window: 1600 ms vs. 1200 ms. It remains to be investigated in future research which task parameter is most crucial for observing or not observing (and if observed the nature of) performance differences in smokers versus non-smokers. A second limitation is the absence of an objective test for compliance with the instruction given to smokers to abstain from smoking for at least 1 h prior to being tested. Without such measure, we cannot be sure whether or not the effects found might be influenced by acute nicotine effects caused by participants who potentially did not comply with the instructions and had smoked just before testing. There was a large range in nicotine dependence in our sample of smokers, as measured by the FTND. This, in principle, allows for an evaluation of the association between level of nicotine dependence and go/no-go performance measures. We performed the corresponding ANOVAs and regression analyses directed at such evaluation and failed to find any significant associations. For example, ANOVAs with Level of Nicotine Dependence Group (3 levels) as factor and the mean go RT, no-go accuracy, and go accuracy data from the 200-ms presentation time condition (pooled across picture type and percentage of no-go pictures) as dependent variables did not reveal any significant Group effect, all *F*s(2, 27) < 1. Likewise, regression analyses with FTND as predictor and each of the same variables as used in the ANOVAs as criterion, also failed to reveal any significant effects, βs < .24, *F*s(1, 28) < 1.67, *p*s > .21. However, the small number of participants included in each level-of-dependence group precludes drawing any clear conclusions on this issue. Another limitation concerns the fact that the present study does not enable us to determine whether the increased response speed observed for the smokers under the more difficult task conditions is caused by smoking or reflects a cognitive or personality feature that is a causal factor for initiating smoking in the first place. Future research should use longitudinal designs to address this causality issue. Further, we only examined male student smokers. Previous studies suggest that there might be gender differences in inhibitory control in general [27], and in terms of differences between smokers and non-smokers in particular [5], and it remains to be investigated whether the increased response speed also holds for female student and non-student samples. A final limitation is the exclusive use of behavioral measures. Future studies should examine neural correlates of the effects found in this study to further specify the nature of the observed effects.

Despite these limitations, the present study contributes to the literature on the relation between smoking and response inhibition. It further specifies the conditions under which smokers may be expected to show impaired response inhibition and may help to solve the cause of the mixed findings of previous research. The present study suggests that such impairments are especially prone to occur under circumstances involving speeded habitual responses. This suggestion may be of value in designing future studies on response inhibition in general, and in enhancing the diagnostic value of the go/no-go task to detect inhibition deficits in specific populations in particular.

## Conclusion

This study contributes to unraveling the conditions under which smokers display impaired performance in go/no-go tasks and helps to characterize the nature of this impairment. Under short stimulus presentation conditions, male student smokers responded faster to go stimuli than non-smokers. This faster responding occurred irrespective of probability of no-go stimuli and of whether the no-go stimuli consisted of cigarette-related or -unrelated pictures. Apparently, especially under task conditions prompting relatively fast responding, compared to non-smokers, smokers are more prone to increase their overall response speed, which is associated with making more errors.

## Supporting Information

S1 Data (SAV)
